# Comparison of Flexural Strength and Flexural Modulus of Conventional and Graphene-Reinforced Polymethyl-Methacrylate under Thermocycled and Non-Thermocycled Conditions: An in-Vitro Study

**DOI:** 10.12669/pjms.42.9.15454

**Published:** 2026-09

**Authors:** Wajiha Zia, Naseer Ahmed, Huda Adil, Rizwan Jouhar

**Affiliations:** 1 Wajiha Zia, Postgraduate Resident, Department of Prosthodontics, Altamash Institute of Dental Medicine, Karachi, Pakistan; 2 Naseer Ahmed, Professor, Department of Prosthodontics, Altamash Institute of Dental Medicine, Karachi, Pakistan; 3 Huda Adil, Postgraduate Resident, Department of Prosthodontics, Altamash Institute of Dental Medicine, Karachi, Pakistan; 4 Rizwan Jouhar, Associate Professor, Department of Restorative Dental Sciences, College of Dentistry, King Faisal University, Al Ahsa, Saudi Arabia

**Keywords:** Flexural strength, Flexural modulus, Graphene, Polymethyl methacrylate, Thermocycling, Three-point bending test

## Abstract

**Background and Objective:**

Polymethyl methacrylate (PMMA) is widely used as a denture base material, but its major disadvantage is its low flexural strength. Graphene has recently been proposed as a reinforcing nanofiller due to its superior mechanical properties, but evidence regarding its incorporation into PMMA and the influence of thermocycling remains limited. This study aimed to evaluate the effect of graphene reinforcement on the flexural strength (FS) and flexural modulus (FM) of PMMA and to assess the impact of thermocycling on these properties.

**Methodology:**

Seventy-six PMMA samples were fabricated and divided into two groups: conventional PMMA (n = 38) and G-PMMA reinforced with 0.25 wt% graphene (n = 38). Each group was further subdivided into thermocycled and non-thermocycled groups (n=19). All samples were subjected to three-point bending tests using a universal testing machine (UTM). Data was analyzed using a two-way ANOVA followed by Bonferroni correction. (p < 0.05).

**Results:**

G-PMMA displayed higher mean FS and FM than conventional PMMA in both thermocycled and non-thermocycled groups. Anova showed a statistically significant difference in flexural strength after thermocycling between PMMA and G-PMMA (p = 0.04), and for flexural modulus, PMMA vs G-PMMA, thermocycling, and their interaction had significant effects (p = 0.043, p = 0.016, and p < 0.001, respectively). Followed by pairwise comparisons, which revealed a statistically significant difference on FS between PMMA and G-PMMA after thermocycling. (p = 0.03)

**Conclusion:**

G-PMMA demonstrated significantly improved flexural strength and modulus compared to conventional PMMA. Thermocycling had no significant effect on flexural strength but influenced flexural modulus, indicating its impact on material stiffness rather than strength.

## INTRODUCTION

People who are partially or completely edentulous require removable prosthesis, which can be manufactured from various materials such as wood, ivory, vulcanite, stainless steel, and polymethyl methacrylate (PMMA), which was invented by Wright W et al.^1^ in 1937. PMMA has gained popularity due to its low cost, adequate mechanical strength, acceptable aesthetics, and good dimensional stability. However, it has low flexural strength, which leads to fracture of the prosthesis.^2^,^3^ Flexural strength is the maximum resistance to fracture that a prosthesis can withstand.^4^ Fracture of the prosthesis is due to masticatory forces, which are the combination of compressive, tensile, and shear stresses; therefore, denture base material should have high flexural strength to resist deformation under these forces.^5^-^7^ In this regard, some studies have shown that 68-70% of dentures fracture within the first three years of their use due to excessive masticatory forces.^2^ It is also important to investigate the impact of intraoral conditions on the mechanical properties of denture-base materials since the oral cavity is a thermodynamic medium that can alter a material’s properties and strength.^8^

In order to improve the strength of a denture, extensive research has been conducted on reinforcing materials such as carbon tubes, metal mesh (Cobalt chromium alloy, stainless steel), BioHPP (biocompatible high-performance polymer), and glass fiber.^9^-^11^ However, they have some drawbacks, like poor aesthetics and recurring fractures.^3^ Hence, to overcome these challenges, graphene was proposed as a promising ingredient to enhance the overall mechanical strength.^12^

Graphene was invented by Novoselov et al.,^13^ which is a two-dimensional, monolayer sp2 hybridized carbon atoms and recognized as the thinnest material. Comparing it to other nanofillers, its tensile strength, flexibility, and low coefficient of thermal expansion give better results.^14^ Recent studies have discovered that graphene and its derivatives have better outcomes than conventional materials.

The study of Di Carlo S et al.^15^ found that the mean value of FS by G-PMMA was greater (113.03 ± 2.94 MPa) than PMMA (96.32 ± 8.78 MPa). Similarly, in a study by De Angelis F et al.,^16^ demonstrated that G-PMMA exhibited the higher mean value for both FS =119.4 MPa and FM =2670.2 MPa as compared to PMMA (FS=113.5 MPa, FM = 2505.3 MPa).

Although extensive research has been conducted on integrating graphene with dental composites, there has been less focus on its incorporation with PMMA.^5^ The primary hypothesis was that there would be no difference in flexural strength and modulus of G-PMMA and conventional PMMA, while the secondary hypothesis was that thermocycling would influence the properties of both materials. Therefore, this study aimed to evaluate the flexural strength and modulus of acrylic resin with the addition of graphene and to determine the effects of thermocycling.

## METHODOLOGY

This in-vitro experimental study was performed at Altamash Institute of Dental Medicine in the Prosthodontic department.This study was reported in accordance with the Checklist for Reporting In-vitro Studies (CRIS) guidelines.^17^ The graphene (Miraculum Graphene Private Limited, India) was reduced at Karachi University’s Food Science Department. The digital metal bar (CoCr, Eplus3D, Hangzhou, China) was fabricated at Chughtai Lab in Peshawar, and the acrylic (Mr. Teeth, Royale Elite, Surrey, U.K) samples were cured at Bahria Dental University, Karachi. Further FS and FM analysis were performed at the research laboratory of Dow University of Health Sciences.

### Ethical Approval:

It was obtained from the Ethical Review Committee (ERC code AIDM/ERC/11/2024/01) on November 28, 2024 and the Research Evaluation Unit (REU code CPSP/REU/DSG-2023-214-4683) of the College of Physicians and Surgeons on 23-4-2025.

A metal bar (65×10×3mm^3^) was fabricated in accordance with ISO 178 Standard^18^ through the selective laser melting technique (SLM) (Audental, Shanghai, China) using a 3D printer. A silicon mold (Lascod Silaxil condensation silicon, Sesto Fiorentino, Italy) was made from a metal bar in which the powder-liquid ratio of PMMA was mixed according to the manufacturer’s guidelines and placed in a doughy stage for the fabrication of the control group. For the experimental group (G-PMMA), 0.25g of graphene was chemically reduced and added to the PMMA powder, then mixed with monomer and packed in a mold. After flask closure, the samples were subjected to bench curing followed by a short curing cycle of two hours at 74°C and one hour at 100°C.^16^ Following polymerization, all the samples were removed, and finished using 220 to 600-grit silicon carbide paper. (Fig.1) All the samples were labeled accordingly. (e.g., PMMA-T, PMMA-NT, G-PMMA-NT, and G-PMMA-T) All the procedures were performed by the principal investigator (W.Z) and the R3 resident (H.A). The coding was concealed from the operator performing the F.S and F.M testing to ensure blinding and minimize biasness.

**Fig.1:**
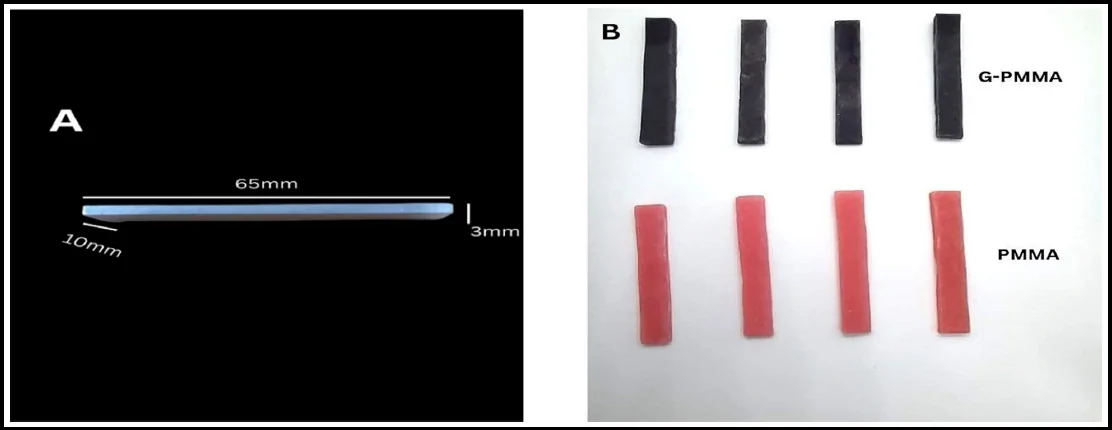
(A) Metal bar (B) G-PMMA and PMMA samples.

A total of 76 samples, 38 samples per group, were then further divided into two subgroups: thermocycled and non-thermocycled, (Fig.2). The sample size was calculated from a previous study in Open-Epi software by using mean flexural strength values of 68.16 ± 5.79MPa for the experimental group and 72.6 ± 7.84^9^ MPa for the control group, with a power of 80%, a confidence interval of 95%, and a margin of error of 5%.

**Fig.2:**
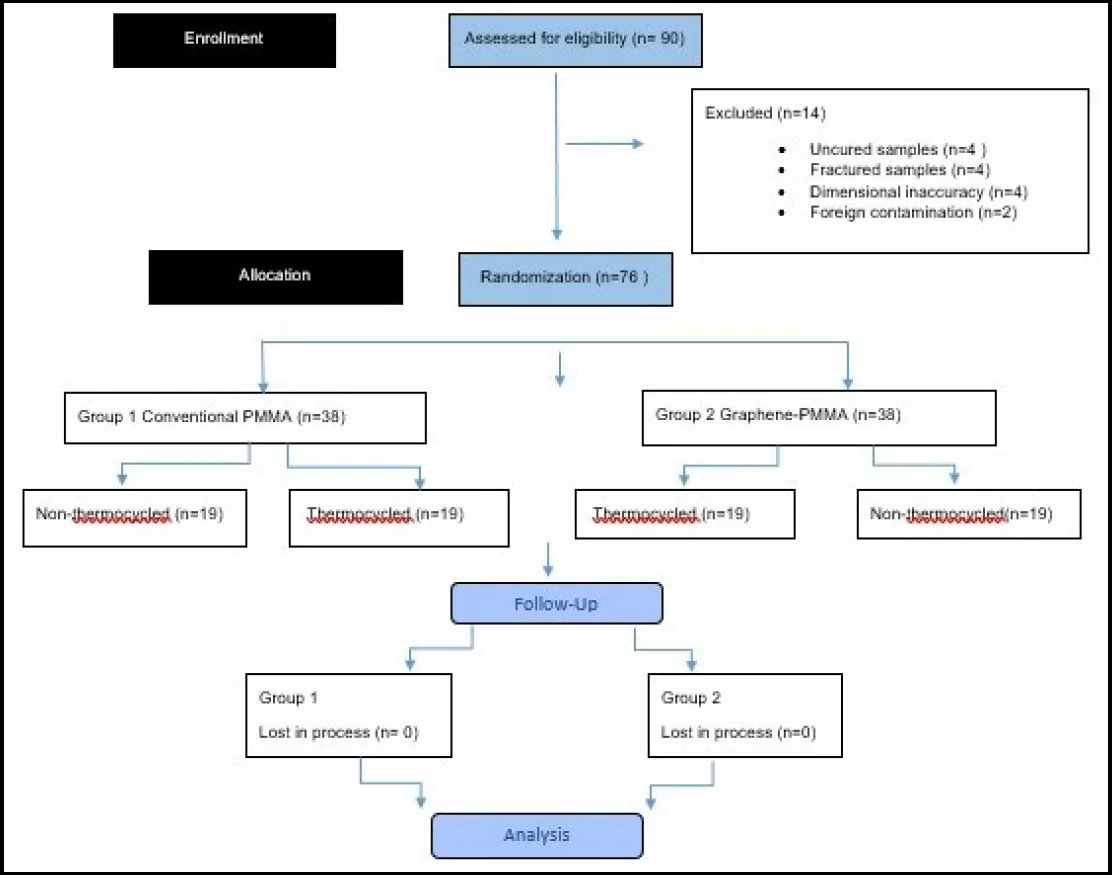
Flow diagram of the study.

Before testing, 19 samples from each group were treated with 2000 cycles in a distilled water bath (thermocycler, San Francisco USA), simulating the two years of clinical aging of denture base materials. The soaking time was 30 seconds at temperatures of 5° and 55° (ISO Standard 11405) using a 15-liter bath with a dwell time of 10 seconds.^8^ Random number generator^19^ (Version 4.0 by Urbaniak, G. C., & Plous, S, 2013) was used to determine the testing sequence of each sample. The FS and FM were tested using a three-point bending test on a UTM (Testometric testing machine, model VB50-300, Rochdale**,** Greater Manchester**,** United Kingdom**)** by applying a load of 0 N at 2mm/min, and the force was increased until the specimens fractured. (Fig.3)

**Fig.3:**
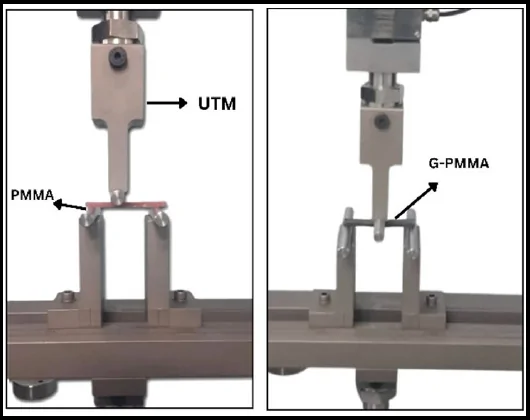
Three-point bending test by universal testing machine (UTM).

In this study, the three constant variables were specimen dimensions (65×10×3mm^3^), thermocycling, and UTM. The dependent variables were flexural strength and modulus, whereas the independent variables were material type (PMMA and G-PMMA), and thermocycling. The accuracy of the UTM machine was calibrated by a trained technician.

### Statistical analysis:

The data was evaluated using SPSS version 26 (SPSS IBM,Chicago, Illinois, U.S). The normality of data was determined by the Shapiro-Wilk test, which showed non-parametric [Flexural strength non-thermocycling control group (FSNTCG), Flexural strength non-thermocycling experimental group (FSNTEG), Flexural strength thermocycling experimental group (FSTEG), Flexural modulus non-thermocycling experimental group (FMNTEG), Flexural modulus thermocycling experimental group (FMTEG)] and parametric [Flexural strength thermocycling control group (FSTCG), Flexural modulus non-thermocycling control group (FMNTCG), Flexural modulus thermocycling control group (FMTCG)] distribution. Mean and standard deviation values were calculated for variables such as flexural strength and modulus subjected to thermocycling and non-thermocycling conditions. A two-way analysis of variance was applied to determine significant differences in F.S and F.M, followed by Bonferroni corrections for multiple pairwise comparisons between groups.(FSNTCG vs FSTCG, FSNTCG vs FSNTEG, FSTCG vs FSTEG, FSTCG Vs FSNTEG, FMNTCG vs FMTCG, FMNTCG vs FMNTEG, FMTCG vs FMTEG,FMTCG vs FMNTEG). The level of significance was set at p < 0.05.

## RESULTS

The result of the study revealed that the mean value of FS was highest in G-PMMA (127.55 ± 43.8 MPa) before thermocycling, followed by FSTEG, FSNTCG, and FSTCG. Furthermore, FM was also higher in G-PMMA (FMNTEG=7472.15 ± 6510.92) before thermocycling as compared to after the thermocycling test. (Fig.4, Table-I)

**Fig.4:**
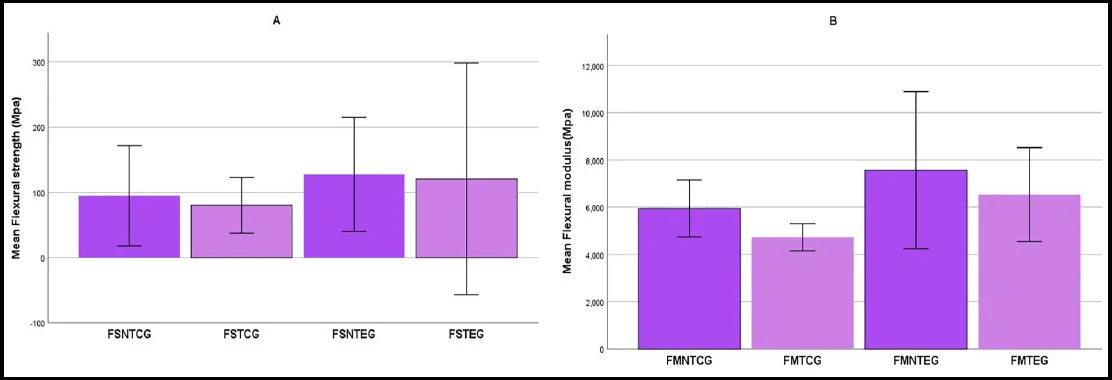
Flexural properties of groups (A) Flexural strength (Mpa) (B) Flexural modulus (Mpa).

**Table-I:** Distribution of Mean ± Standard deviation of FS and FM in experimental and control groups (n=76).

Groups	Mean±Std (MPa)
FSNTCG	94.67 ± 38.53
FSTCG	80.14 ± 21.36
FMTCG	4770.75 ± 1239.76
FMNTCG	5849.17 ± 2542.76
FSNTEG	127.55 ± 43.8
FSTEG	120.58 ± 88.79
FMNTEG	7472.15 ± 6510.92
FMTEG	6534.37 ± 4126.43

Material type (PMMA vs G-PMMA) had a statistically significant effect on flexural strength (F = 8.69, p = 0.04), indicating a difference between the two materials, while thermocycling (p = 0.39) and the interaction between material and thermocycling (p=0.762) showed no significant influence. For flexural modulus, material type (F = 3.23, p = 0.043), thermocycling (F = 1.14, p = 0.016), and their interaction (p = 0.001) were all statistically significant, demonstrating that both the material and thermal aging affect stiffness, and that the impact of thermocycling varies depending on the material (Table-II).

**Table-II:** Comparison of flexural properties between PMMA and G-PMMA under thermocycling conditions (n=76)

Variable	Comparison/variation	df	f	p-value
Flexural strength	Materials(PMMA vs G-PMMA)	1	8.69	0.04*
	Thermocycling	1	0.748	0.39
	Materials×Thermocycling	1	0.092	0.762
Flexural modulus	Materials(PMMA vs G-PMMA)	1	3.23	0.043*
	Thermocycling	1	1.14	0.016*
	Materials×Thermocycling	1	0.006	0.00*

*Statistically significant at p < 0.05.

Table-III showed pairwise comparisons, which revealed a statistically significant difference (p=0.03) between FSTCG vs FSTEG and FSTCG vs FSNTEG (p = 0.01), while all other comparisons were not statistically significant (p ≥0.05).

**Table-III:** Post-hoc analysis of flexural strength between thermocycled and non-thermocycled groups

Comparison	Mean Difference	p-value
FSNTCG vs FSTCG	14.53	0.78
FSNTCG vs FSNTEG	32.88	0.08
FSTCG vs FSTEG	40.44	0.03*
FSTCG vs FSNTEG	47.41	0.01*
FMNTCG vs FMTCG	1078.42	0.82
FMNTCG vs FMNTEG	1622.98	0.52
FMTCG vs FMTEG	1763.62	0.43
FMTCG vs FMNTEG	2701.40	0.23

*Statistically significant at p < 0.05.

## DISCUSSION

The present study investigated the comparison of flexural strength and modulus of PMMA with graphene-reinforced PMMA, and the impact of thermocycling. The null hypothesis was rejected, indicating a significant difference in flexural strength between groups. However, the secondary hypothesis was partially rejected as the effect of thermocycling was less in G-PMMA as compared to PMMA. The results showed that before thermocycling, G-PMMA had the highest FS and FM as compared to conventional PMMA.

Adequate flexural strength is essential for denture performance, as masticatory forces can initiate crack propagation, which leads to failure of the prosthesis. The addition of 0.25g of graphene improved the mechanical properties of denture base material, which showed a statistically significant difference was observed only between FSTCG and FSTEG (p = 0.03) and between FSTCG vs FSNTEG (p = 0.01); however, no statistically significant difference was observed among the other groups (p ≥0.05). In contrast, Bacali et al. reported that 74% improvement in flexural strength was observed following the incorporation of 2% silver graphene (Gr-Ag) into PMMA resin.^3^ The reason for this difference may be attributed to the lower concentration of graphene (0.25wt%) in the present study. Previous literature has reported graphene loadings ranging from 0.5 to 20%.^3^,^20^-^21^ However, higher concentrations are often associated with nanoparticle agglomeration, whereas some studies suggested that low filler concentrations may yield more favorable outcomes due to improved dispersion. For instance, a study reported superior mechanical properties with only 0.01% graphene as compared to the control group.^22^ Similarly, Ramanathan et al demonstrated that the addition of 1% of graphene to PMMA leads to an increase of 80% elastic modulus and a 20% increase in tensile strength.^23^ Moreover, in the present study, graphene was incorporated in its reduced form as a standalone filler, whereas several other studies have combined graphene with other nanoparticles such as silver or hydroxyapatite, which may contribute to greater improvements in mechanical properties 3,21

In this study, the powder form of graphene was incorporated into the polymer, resulting in a slight increase in flexural properties. Some variability in the results was observed in a certain group, particularly in graphene-reinforced samples, which indicates uneven distribution of graphene within the PMMA matrix. In contrast, Ghosh M et al.^12^ reported a reduction in F.S when 0.5wt% graphene was added into the monomer. This discrepancy may be due to graphene sheets agglomerating in monomers, forming larger particles with a small surface area. This causes steric hindrance, which prevents further chemical interactions that block polymer flow. Hence, it promotes void formation that serves as stress concentration sites and increases the risk of mechanical failure.^13^ Therefore, the variation between studies may be related to differences in the method of graphene incorporation, as the current study achieved more homogeneous mixing by adding graphene directly to the polymer.

The deterioration in the mechanical performance of denture base material during clinical use can also be attributed to thermal fluctuations and water sorption, which lead to polymer degradation and reduce strength.^8^ The result of this study indicates that both PMMA and G-PMMA had a significant effect of thermocycling on both FS and FM. Furthermore, the interaction between materials and thermocycling was not significant for FS, indicating that the effect of graphene reinforcement remained consistent regardless of thermal aging. In contrast to the finding of Temizci T et al.^24^ who reported a significant effect when material and thermocycling were evaluated. In contrast, thermocycling showed a statistically significant effect on FM, which suggests that thermal aging may alter the stiffness of the material without markedly affecting its resistance to fracture. These findings are consistent with the study of Segerström et al.^25^ who observed a higher FM of PMMA following thermal cycling. This study indicates that thermocycling resulted in a reduction of both F.S and F.M in control and experimental groups. However, the control group exhibited a 1.5-times greater reduction in F.S and a 1.4-times greater reduction in flexural modulus compared with the experimental group. Pre-thermocycling, G-PMMA demonstrated superior mechanical properties, with FS and FM about 1.5 times and 1.3 times greater than experimental group.

### Strength

The study’s strength is that it contributes valuable information to global medical and dental literature by introducing 0.25% graphene, based on previous literature suggesting that lower concentrations may provide a reinforcement effect on the flexural properties of PMMA, while maintaining low-cost effectiveness and minimizing potential issues related to nanoparticle agglomeration.

### Limitations

It includes the fabricated specimens did not replicate a complete denture, and thermal cycling was performed in distilled water instead of an artificial saliva. Additionally, the dark color of graphene may also restrict its application in the esthetic zones of dentures.

## CONCLUSIONS

Within the limitations of the present study, it was concluded that G-PMMA demonstrated significantly improved flexural strength and modulus compared to conventional PMMA. Thermocycling had no significant effect on flexural strength but influenced flexural modulus, indicating its impact on material stiffness rather than strength. Overall, G-PMMA exhibits superior and more reliable mechanical properties, supporting its potential as a clinically favorable alternative to conventional PMMA for improved durability under simulated oral conditions. However, further in-vivo studies are recommended to confirm these findings before clinical application can be considered.

### Recommendations

Future studies should focus on improving nanoparticle dispersion methods and standardizing fabrication protocols to minimize variability and enhance reproducibility. Moreover, further investigation should address the discoloration issue, optimal concentration of graphene, and investigate full denture base specimens to more accurately reflect clinical conditions and predict long-term performance.

### Author’s Contribution:

**WZ:** Study conception and design, data collection, and writing manuscript. Responsible and accountable for the accuracy and integrity of the work. **NA, RJ:** Analysis and interpretation of results and assistance and interpretation, Supervision, Critical review. **HA:** Manuscript revision and validation. All authors have read and approved the final version of the manuscript.
